# Supplementary figures and images for: Proteome Analysis Reveals Syndecan 1 Regulates Porcine Sapelovirus Replication
Source: Int J Mol Sci. 2020 Jun 19;21(12):4386. doi: 10.3390/ijms21124386 (PMC7352226; doi:10.3390/ijms21124386)

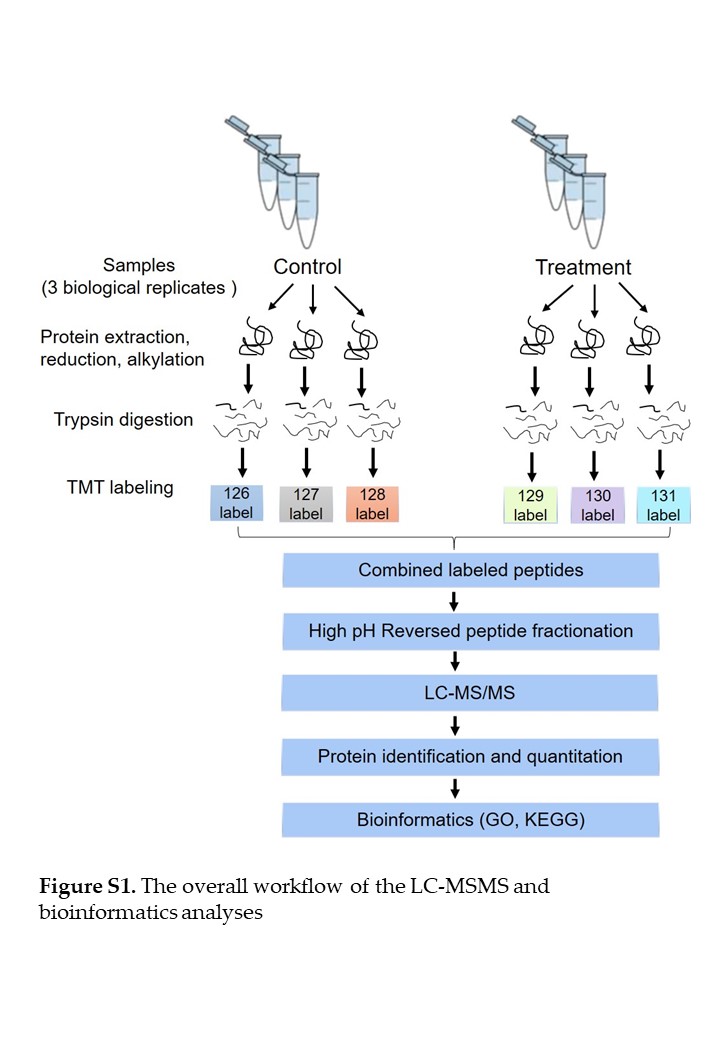

Supplement: Supplementary file 1 [file ijms-21-04386-s001.zip › Supplementary materials/Figure S1.jpg]
