# Supplementary material for: Proteome Analysis Reveals Syndecan 1 Regulates Porcine Sapelovirus Replication
Source: Int J Mol Sci. 2020 Jun 19;21(12):4386. doi: 10.3390/ijms21124386 (PMC7352226; doi:10.3390/ijms21124386)
Supplement: Supplementary file 1 [file ijms-21-04386-s001.zip › Supplementary materials/Table S8.doc]

Table S8. Primer sequences of Real Time qPCR.

| name | primer (5’-3’) |
| --- | --- |
| SPP1-F | GAGCAAACAGACGATGTGGA |
| SPP1-R | GAAATCGGTGACCAGCTCAT |
| IFIT5-F | CCGTTATGCAGCCAAATTCT |
| IFIT5-R | TCTGTTTCGAGTGGCCTTCT |
| ISG15-F | GGTGCAAAGCTTCAGAGACC |
| ISG15-R | CCTCGAAAGTCAGCCAGAAC |
| SDC1-F | ACCCACTCACCCAGTGAGAC |
| SDC1-R | ACCAGGAGTGTGAGGGTCAA |
| VCL-F | CAGACCTGCTCCTCACCTTC |
| VCL-R | TGGTGAGTCAGTTCCTGCTG |
| GAPDH-F | TTTAACTCTGGCAAAGTGGACA |
| GAPDH-R | GGCCTTTCCATTGATGACAAGC |
